# Licochalcone a Exhibits Leishmanicidal Activity *in vitro* and in Experimental Model of *Leishmania* (*Leishmania*) *Infantum*

**DOI:** 10.3389/fvets.2020.00527

**Published:** 2020-12-10

**Authors:** Julia M. Souza, Érica A. A. de Carvalho, Ana Carolina B. B. Candido, Rafael P. de Mendonça, Maria Fernanda da Silva, Renato L. T. Parreira, Fernanda G. G. Dias, Sérgio R. Ambrósio, Andrea T. Arantes, Ademar A. da Silva Filho, Aline N. Nascimento, Monique R. Costa, Mirela I. Sairre, Rodrigo C. S. Veneziani, Lizandra G. Magalhães

**Affiliations:** ^1^Núcleo de Pesquisa em Ciências Exatas e Tecnológica, Universidade de Franca, Franca, Brazil; ^2^Pós Graduação em Ciência Animal, Universidade de Franca, Franca, Brazil; ^3^Centro Tecnológico Agropecuário, Batatais, Brazil; ^4^Departamento de Ciências Farmacêuticas, Universidade Federal de Juiz de Fora, Juiz de Fora, Brazil; ^5^Centro de Ciências Naturais e Humanas, Universidade Federal Do ABC, Santo André, Brazil

**Keywords:** cutaneous leishmaniasis, visceral leishmaniasis, canine visceral leishmaniasis, licochalcone A, leishmanicidal activity

## Abstract

The efficacy of Licochalcone A (LicoA) and its two analogs were reported against *Leishmania (Leishmania) amazonensis* and *Leishmania (Leishmania) infantum in vitro*, and in experimental model of *L. (L.) infantum in vitro*. Initially, LicoA and its analogs were screened against promastigote forms of *L. (L.) amazonensis*. LicoA was the most active compound, with IC_50_ values of 20.26 and 3.88 μM at 24 and 48 h, respectively. Against amastigote forms, the IC_50_ value of LicoA was 36.84 μM at 48 h. In the next step, the effectivity of LicoA was evaluated *in vitro* against promastigote and amastigote forms of *L. (L.) infantum*. Results demonstrated that LicoA exhibited leishmanicidal activity *in vitro* against promastigote forms with IC_50_ values of 41.10 and 12.47 μM at 24 and 48 h, respectively; against amastigote forms the IC_50_ value was 29.58 μM at 48 h. Assessment of cytotoxicity demonstrated that LicoA exhibited moderate mammalian cytotoxicity against peritoneal murine macrophages; the CC_50_ value was 123.21 μM at 48 h and showed about 30% of hemolytic activity at concentration of 400 μM. *L. (L.) infantum-*infected hamsters and treated with LicoA at 50 mg/kg for eight consecutive days was able to significantly reduce the parasite burden in both liver and spleen in 43.67 and 39.81%, respectively, when compared with negative control group. These findings suggest that chalcone-type flavonoids can be a promising class of natural products to be considered in the search of new, safe, and effective compounds capable to treat canine visceral leishmaniosis (CVL).

## Introduction

Leishmania parasites are the etiological agents that cause leishmaniasis, and the parasites are transmitted by the bite of infected female phlebotomine sandflies (1). Human disease is classified into three main clinical forms: cutaneous leishmaniasis (CL), mucocutaneous leishmaniasis (MCL), and visceral leishmaniasis (VL) (1). CL is the most common form of the disease, and the manifestations can depend of the parasite species and host immune responses (2). *Leishmania (Viannia) braziliensis* and *Leishmania (Leishmania) amazonensis* are the main species responsible by CL in Brazil, and also by diffuse cutaneous leishmaniasis (DCL) (2). On the other hand, VL exists in two forms, zoonotic and anthroponotic, which are caused by *Leishmania (Leishmania) infantum* and *Leishmania (Leishmania) donovani*, respectively (3). Among the different mammal species, dogs can be considered as the main reservoirs of *L. (L.) infantum*, and this parasite also is responsible by canine visceral leishmaniasis (CVL) (4). Due the extension of CVL in urban regions in South American countries, this parasite has the attention of public health and the scientific community (4, 5).

The chemotherapy of leishmaniasis on human depends on three drugs, pentavalent antimonials, such as meglumine antimoniate (MA), amphotericin B (AmpB), and miltefosine (6). These drugs have high cost, limited efficacy, long administration protocols, severe side-effects, and development of drug resistance upon repeated use. In relation on CLV, miltefosine is an alternative for treatment of animals in regions where this drug is not used in humans; however, studies shown that miltefosine reduces the parasitic load of dogs infected with *L. (L.)infantum*, but no parasitological cure (4, 7, 8).

**Figure 1:**
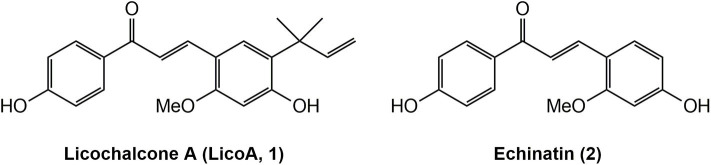
Structures of natural products Licochalcone A (LicoA **1**) and Echinatin (**2**).

Licochalcone A (LicoA, **1**) (Figure 1) is a naturally occurring chalcone-type flavonoid isolated from roots of the Chinese licorice (*Glycyrrhiza inflata*) that can be considered an antiparasitic hit compound (9). It can chemically describe as an α,β-unsaturated bisphenylic ketone (chalcone skeleton) substituted with two phenolic hydroxyl groups, one methoxy moiety, and an isoprenoid side chain. Previous works described the significant *in vitro* and *in vivo* effect of LicoA against *Leishmania (L.) major* and *L. (L.) donovani*, which are, respectively, associated with the CL and VL forms (10, 11). Another chalcone-type flavonoid compound from licorice roots extracts is Echinatin (**2**) (Figure 1). It has been reported to have a broad range of bioactivities, such as hepatoprotective (12) anti-inflammatory, antioxidant (13, 14), antimicrobial (15), and cardioprotective (16).

**Figure 2:**
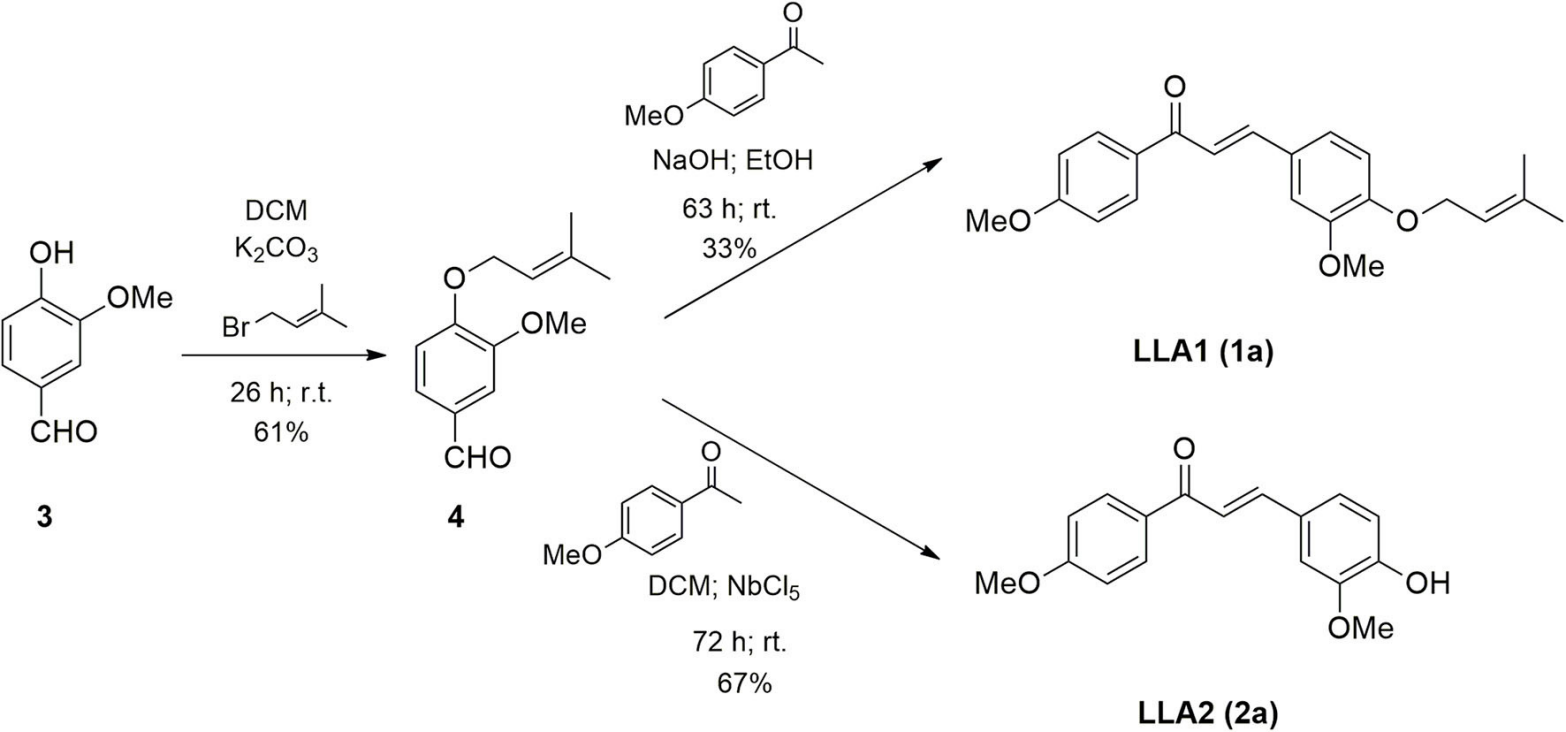
Scheme of synthesis of LLA1 **(1a)** and LLA2 **(1a)**.

Considering that LicoA has demonstrated its potential against other forms of the parasite and that other analogs of LicoA could also present promising results, the aim of this study is to evaluate the *in vivo* leishmanicidal effect of LicoA and its two analogs against *L. (L.) amazonensis* and *Leishmania (L.) infantum in vitro* and in an experimental model of *Leishmania (Leishmania) infantum* in hamster. The analogs were synthesized and constitute structures similar to the natural products LicoA (**1**) and also Echinatin (**2**) (Figure 1). The analog LLA1 (**1a**) (Figure 2) is a derivate of LicoA that belongs to the class of chalcones containing the group *O*-prenil (17). In another study, the evaluation of a series of prenyloxy and geranyloxy chalcones against *Leishmania (Leishmania) mexicana* and *Trypanosoma cruzi* demonstrated that the position of the substituent has an influence on the activity and selectivity of these compounds (18). The antiparasitic activity of the analog LLA2 (**2a**) (Figure 2) has not been investigated yet.

## Materials and Methods

### Reagents and Compounds

The reagents and solvents used in the synthesis of the analogs of LicoA (LLA1 **1a** and LLA2 **2a**) were obtained from commercial sources and used directly without further purification. NbCl_5_ was supplied by Companhia Brasileira de Metalurgia e Mineração (CBMM). Thin layer chromatography (TLC) was performed on Sigma-Aldrich silica gel matrix, pre-coated plates with fluorescent indicator 254 nm (Sigma-Aldrich, St Louis, USA). ^1^H and ^13^C NMR spectra were recorded on Varian equipment (500 MHz) from Federal University of ABC or Bruker equipment (DPX-300 and DPX-400) from University of São Paulo, Ribeirão Preto-SP, using the solvent deuterated chloroform (CDCl_3_) or dimethylsulfoxide (DMSO-d6). HRMS were obtained in instrument micrOTOFfrom University of São Paulo, Ribeirão Preto-SP. AmpB was purchased from Sigma-Aldrich, and MA (Glucantime®) was obtained from Sanofi-Aventis (Sanofi-Aventis, São Paulo, Brazil), which each 5 mL of aqueous solution contained 1.5 g of MA and represented 405 mg of pentavalent antimonial (Sanofi-Aventis).

### Chemistry

#### Isolation of LicoA (1)

The isolation and identification of LicoA was performed as previously described (19) and its chemical structure was identified by ^1^H- and ^13^C-NMR analysis data in comparison with the literature (20). Purity of LicoA (**1**) was estimated to be higher than 95% by both ^13^C NMR and HPLC analysis using different solvent systems.

#### Synthesis of Compound LLA1 (1a) and LLA2 (2a) (Scheme 2)

##### 3-methoxy-4-((3-methylbut-2-en-1-yl)oxy)benzaldehyde (4)

The potassium carbonate was previously macerated with a mortar and pistil. Vanillin (**3**) (0.4526 g; 3 mmol), potassium carbonate (1.6970 g; 12 mmol), prenyl bromide (0.353 mL, 3 mmol), tetrabutyl ammonium chloride (0.0816 g; 0.3 mmol), and dichloromethane (DCM) were added to a round-bottom flask. The reaction was kept under stirring and at room temperature. The reaction was monitored by TLC until the practically total consumption of vanillin was verified (26 h). Then, the material was dissolved in dichloromethane and transferred to a separating funnel. The organic layer was separated and the aqueous layer extracted twice with dichloromethane. The combined organic extracts were washed with brine and the solution obtained was dried over anhydrous magnesium sulfate, filtered, and the solvent was evaporated under reduced pressure. The crude product obtained was purified over silica gel column chromatography using the mixture of 80% *n*-hexane in ethyl acetate as eluent to yield 0.4007 g of the pure product (61%) of **4**. ^**1**^**H-RMN (CDCl**_**3**_**, 300 MHz)** **δ:** 1.75 (d, 3H, *J* = 1.2 Hz); 1.80 (d, 3H, *J* = 1.2 Hz); 4.58 (d, 2H. *J* = 6.7 Hz); 5.48 (tsept, 1H, *J* = 6.7 Hz; *J* = 1.2 Hz); 7.00 (d, *J* = 8.7 Hz); 7.82 (d, *J* = 8.7 Hz); 9.87 (s, 1H). ^**13**^**C-RMN (CDCl**_**3**_**, 75 MHz)** **δ:** 18.18 (CH_3_), 25.72 (CH_3_); 65.17 (CH_2_); 114.95 (CH); 118.87 (CH); 129.86 (C); 131.88 (CH); 138.89 (C); 163.98 (C), 190.64 (CH).

##### (E)-3-(3-methoxy-4-((3-methylbut-2-en-1-yl)oxy)phenyl)-1-(4-methoxyphenyl)prop-2-en-1-one (LLA1 1a)

To a round-bottom flask, sodium hydroxide (0.0462 g; 0.9 mmol), ethanol (4 mL), and *p*-methoxyacetophenone (0.0754 g; 0.45 mmol) were added. The mixture was kept in an ice bath for 20 min. Then, the prenylated vanillin (**4**) obtained in the previous reaction (0.1001 g; 0.45 mmol) was added and the temperature was spontaneously raised to room temperature. The reaction was kept under stirring at room temperature until the total reagent consumption was observed by TLC (63 h). Ethanol was removed under reduced pressure. Then the material was dissolved in ethyl acetate and transferred to a separatory funnel. The aqueous phase was extracted twice with ethyl acetate. The combined organic phases were washed with brine. The organic extract was dried over anhydrous magnesium sulfate, filtered, and the solvent was evaporated under reduced pressure. The crude product obtained was purified over silica gel column chromatography using the mixture of 30% ethyl acetate in *n*-hexane as the eluent to yield 0.0498 g of the pure product (33%) of **1a** as a yellow powder. ^**1**^**H-RMN (CDCl**_**3**_**, 500 MHz)** **δ:** 1.76 (d, 3H, *J* = 1.0 Hz); 1.80 (d, 3H, *J* = 1.0 Hz); 3.89 (s, 3H); 3.94 (s, 3H); 4.64 (d, 2H, *J* = 6.8 Hz); 5.52 (thept, 1H. *J* = 6.8 Hz; *J* = 1.0 Hz); 6.90 (d, 1H, *J* = 8.2 Hz); 6.99 (d, 2H, *J* = 8.8 Hz); 7.17 (d, 1H, *J* = 2.1 Hz); 7.21 (dd, 1H, *J* = 8.2 Hz; *J* = 2.1 Hz); 7.41 (d, 1H, *J* = 15.6 Hz); 7.76 (d, 1H, *J* = 15.6 Hz); 8.04 (d, 2H, *J* = 8.8 Hz). ^**13**^**C-RMN (CDCl**_**3**_**, 125 MHz)** **δ:** 18.28 (CH_3_); 25.84 (CH_3_); 55.47 (CH_3_); 55.97 (CH_3_); 65.77 (CH_2_); 110.27 (CH); 112.58 (CH); 113.77 (CH); 119.42 (CH); 119.66 (CH); 122.87 (CH); 127.91 (C); 130.71 (CH); 131.34 (C); 138.19 (C); 144.27 (CH); 149.55 (C); 150.62 (C); 163.25 (C); 188.81 (C).

##### (E)-3-(4-hydroxy-3-methoxyphenyl)-1-(4-hydroxyphenyl)prop-2-en-1-one (LLA2 2a)

To a round bottom flask fitted with a drying tube were added niobium pentachloride (0.8998 g; 1.35 mmol) and dichloromethane (10 mL). Then, p-methoxyacetophenone (0.1346 g, 0.9 mmol) and prenylated vanillin (0.2085 g, 0.9 mmol) were added. The reaction mixture was stirred at room temperature for 72 h. After the period, an extraction with dichloromethane was carried out, then the solvent was eliminated under reduced pressure and the crude residue was purified over silica gel column chromatography using the mixture of n-hexane and ethyl acetate (2:1) as eluent to yield 0.1814 g (67%) of **2a**. ^**1**^**H NMR (CDCl3, 500 MHz)** δ: 3.87 (s, 3H); 3.93 (s, 3H); 6.95 (d, 1H, J = 8.3 Hz); 6.97 (d, 2H, J = 9.0 Hz); 7.12 (d, 1H, J = 1.9 Hz); 7.20 (dd, 1H, J = 1.9 Hz; J = 8.3 Hz); 7.39 (d, 1H, J = 15.4 Hz); 7.74 (d, 1H, J = 15.4 Hz); 8.03 (d, 2H, J = 9.0 Hz). ^**13**^**C NMR (CDCl3, 125 MHz)** **δ:** 55.46 (CH3); 55.97 (CH3); 110.11 (CH); 113.78 (CH); 114.92 (CH); 119.42 (CH); 123.16 (CH); 127.59 (C); 130.73 (CH); 131.27 (C); 144.46 (CH); 146.88 (C); 148.22 (C); 163.28 (C); 188.90 (C). HRMS m/z [M+H]+ calcd: 285.1121; found: 285.1124.

## Animals

Male Balb/c mice (*Mus musculus*) were acquired from the animal houses of the University of São Paulo (Ribeirão Preto, São Paulo, Brazil) and male golden hamsters (*Mesocricetus auratus*) (110 g) were acquired from ANILAB–Laboratory Animal Creation and Trade Ltd. (Paulínia, São Paulo, Brazil). The experiments were conducted in accordance with the Brazilian legislation regulated by the National Council for the Control of Animal Experimentation (CONCEA) and approved by the University of Franca's Ethics Committee for Animal Care under protocol number 046/15 (Approval Date: November 09, 2015).

## Parasites and Mammalian Cell Maintenance

*L. (L.) amazonensis* (IFLA/BR/67/PH8.) was maintained as promastigote forms in Roswell Park Memorial Institute medium (RPMI 1640 medium) (Gibco, Grand Island, NY, USA), supplemented with antibiotics (penicillin 10.000 UI/mL and streptomycin 10 mg μg/mL) (Cultilab, Campinas, BR), and 10% bovine fetal serum (FBS) (Cultilab), at 25°C. *L. (L.) infantum* (MHOM/BR/1972/LD) was maintained as promastigote forms in M199 medium (Gibco), supplemented with 10% FBS (Cultilab), antibiotics (Cultilab), 5% human urine and 0.25% hemin (Sigma-Aldrich) at 25°C, and amastigote forms were obtained from the spleen of golden hamsters after 60 to 70 days of infection (21). Peritoneal murine macrophages were obtained by washing the peritoneal cavity of BALB/c mice with RPMI 1640 medium (Gibco) after 72 h of the application of 5 mL of the sodium thioglycolate (Sigma-Aldrich) at 3%, and then were cultivated in RPMI 1640 medium (Gibco) supplemented with antibiotics and 10% FBS (Gibco), at 37°C in a 5% CO_2_ humidified incubator.

## *In vitro* Leishmanicidal Activity

Initially, a screening was performed against promastigote forms of *L. (L.) amanzonensis*. Briefly, promastigotes (1 × 10^6^ parasites/well) were distributed in 96-well plates, and the compounds LicoA (**1**), LLA1 (**1a**), LLA2 (**2a**), and MA were added at concentrations 0.78–400.0 μM, and AmpB was added at concentrations 0.0025–1.56 μM. The plates were incubated at same conditions previously described during 48 h, and the leishmanicidal activity was determined by the inhibition of growth of the promastigote forms by counting the total number of live promastigote in Neubauer's chamber (Global Glass, São Paulo, BR) using an optical microscope (Nikon New York, USA). The compound (LicoA, **1**) that exhibited activity against promastigote forms of *L. (L.) amazonensis* also was evaluated against promastigote forms *of L. (L.) infantum* as previously described and against amastigote forms of both *Leishmania* parasites. To evaluate the leishmanicidal activity against amastigote forms, peritoneal murine macrophages were seemed (2 × 10^5^ cells per well) in 24-well plates on slide chambers and incubated at 37°C for 24 h. After the 24 h, the cells were infected with promastigote forms of *L. (L.) amazonensis*, previously acidified (22) or with amastigote forms of *L. (L.) infantum* at a ratio of 10:1 (parasites/macrophage) for 4 h, and subsequently the cells were incubated with the LicoA (**1**) (6.25–100 μM), AmpB (0.095–0.005 μM), or MA (12.5–400 μM) 48 h. The slides were stained with Giemsa (Synth, Diadema, BR), analyzed using an optical microscopy (Nikon), and the parasite load was defined by the number of infected macrophages X number of intracellular amastigotes/number of total macrophages (23). Parasites cultured in medium with 0.1% were used as negative control and parasites cultured in medium with MA or AmpB were used as positive controls.

## *In vitro* Cytotoxic Activity

Peritoneal murine macrophages were seemed (2 × 10^5^ cells per well) in 96-well plates and LicoA (**1**), AmpB, and MA were added at concentrations 12.5–400 μM. The cells were incubated at 37°C in a 5% CO_2_ humidified incubator at 37°C during 48 h, and the cytotoxic activity was determined using MTT assay (23). Cells cultured in medium with 0.1% DMSO were used as negative control and cells cultured in medium with 25% DMSO were used as positive control.

## *In vitro* Hemolytic Activity

Defibrinated sheep blood (Newprov, Pinhais, PR, BR) was diluted in 0.9% saline solution and a suspension at 3% of erythrocytes was transferred (100 μL) in 96-well plates. LicoA (**1**) and AmpB were added (12.5–400 μM), and the plates were incubated for 30 min at 37°C. The hemolytic activity was determined in the cell supernatant by optical density reading at 415 nm (Libra S12–Biochrom–Cambridge, RU) Distilled water was used as a positive control, and 0.9% saline solution with 0.1% DMSO was used as negative control.

## *In vivo* Leishmanicidal Activity of LicoA

The *in vivo* leishmanicidal activity of LicoA (**1**) was assessment in experimental model of *Leishmania (L.) infantum* and conducted as described by (21) with adaptations. Male golden hamsters were infected by intraperitoneal route with 1.0 × 10^8^ *L. (L.) infantum* amastigotes. Four weeks after infection, the animals were randomly separated in 4 groups of 6 animals each. Two groups of treated animals received intraperitoneal doses of 20 and 50 mg/kg of body weight of LicoA (**1**), respectively, for eight consecutive days. Negative control group received the same number of injections of phosphate buffered saline (PBS) and animals from positive control group was treated with MA (Sanofi-Aventis) at dose 50 mg/kg of body weight during eight consecutive days by intraperitoneal route. Thirty five days after treatment the animals were sacrificed and the parasite burden was evaluated both in spleen and liver by limiting dilution method (24).

In order to evaluate the hepatotoxicity, groups with 6 male golden hamsters no-infected were treated intraperitoneally with LicoA (**1**) (20 mg/kg or 50 mg/kg of body weight per day) or MA (50 mg/kg of body weight per day) for 15 days. After this period, serum concentrations of alanine aminotransferase (ALT) and aspartate aminotransferase (AST) were determined using sets of commercial reagents (Labtest, Minas Gerais, Brazil). Negative control group received the same number of injections of PBS.

## Statistical Analysis

The analyses were performed using GraphPad Prism 7 program (GraphPad Software, San Diego, CA, USA). Both IC_50_ and CC_50_ values were calculated with the aid of sigmoid dose–response curves, and the selectivity index (SI) values were determined by CC_50_/IC_50_. *In vitro* experiments were performed in triplicate and repeat two times. *In vivo*, statistical analysis was performed using one-way ANOVA followed by Dunnett's multiple comparison test.

## Results

### Synthesis of LLA1 (1a) and LLA2 (2a)

According to Figure 2, the chalcone containing *O*-prenyl (LLA) was synthesized in two steps. The vanillin prenylation reaction was carried out under conditions of phase transfer catalysis (PTC) that allows the use of aqueous potassium carbonate solution and a transfer catalyst (25). The most commonly used catalysts for this type of reaction are quaternary ammonium salts. Tetrabutyl ammonium chloride (TBAC) was used because it has large substituent groups (*n*-butyl) that favor the reaction. Despite the long reaction time, the procedure was simple, at room temperature and product **4** was obtained with a yield of 61%. The prenylated chalcone (LLA1) (**1a**) was prepared via the Claisen-Schmidt condensation between the prenylated vanillin and commercial *p*-methoxyacetophenone. The reaction was carried out under basic conditions and the yield of the product LLA1 (**1a**) was 33% after purification. The synthesis of LLA2 (**2a)** was carried out through the Claisen-Schmidt condensation by acid catalysis (Figure 2). The reaction between the prenylated vanillin and the *p*-methoxyacetophenone promoted the formation of chalcone, together with the hydrolysis of the prenyl group in the presence of Lewis acid (niobium pentachloride), providing the product **2a** with 67% yield, after purification by chromatography on silica gel column. The use of niobium pentachloride as an acid catalyst is an alternative for the synthesis of chalcones through Claisen-Schmidt.

### *In vitro* Leishmanicidal Activity

Initially, the leishmanicidal activity of LicoA (**1**) and analogs LLA1 (**1a**) and LLA2 (**2a**) was evaluated against promastigotes, and the active compound was subsequently evaluated against amastigote forms of *L. (L.) amazonensis*. LicoA (**1**) exhibited promising leishmanicidal activity *in vitro* with IC_50_ values of 20.26 and 3.88 μM at 24 and 48 h, respectively, against promastigote and IC_50_ value of 36.84 against amastigote forms of *L (L.) amazonensis*. LLA1 (**1a**) was the second most active against promastigote forms with IC_50_ values of 74.94 and 67.16 μM at 24 and 48 h, and LLA2 (**2a**) exhibited modest activity *in vitro* against promastigote forms of *L. (L.) amazonensis* (IC_50_ values > 300 μM). AmpB, used as positive control against *L. (L.) amazonensis*, exhibited IC_50_ values against promastigote forms of 0.27 and 0.065 μM at 24 and 48 h, respectively; and against amastigote forms the IC_50_ value was 0.022 at 48 h. As LicoA (**1**) was the most active, in the next step, the effectivity of LicoA (**1**) was evaluated *in vitro* against promastigote and amastigote forms of *L. (L.) infantum*. LicoA (**1**) exhibited leishmanicidal activity *in vitro* against promastigote forms with IC_50_ values of 41.10 and 12.47 μM at 24 and 48 h, respectively; against amastigote forms the IC_50_ value was 29.58 μM after 48 h. MA, used as positive control exhibited IC_50_ values > 400 μM in both promastigote and amastigote forms (Table 1).

**Table 1:** Leishmanicidal activity and mammalian cytotoxicity of LicoA and its analogs.

**Compounds**	**IC_50_-μM^a^ (95% CI)^b^*L (L.) amazonensis***			**IC_50_-μM^a^ (95% CI)^b^*L (L.) infantum***			**CC_50_-μM^c^ (95% CI)^b^ Peritoneal murine macrophages**
	**Promastigote**		**Amastigote**	**Promastigote**		**Amastigote**	
	**24 h**	**48 h**	**48 h**	**24 h**	**48 h**	**48 h**	**48 h**
LicoA (1)	20.26 (17.26–24.54)	3.88 (2.84–4.89)	36.84 (32.25–40.02)	41.10 (37.55–44.98)	12.47 (10.02–15.06)	29.58 (26.03–33.06)	123.10 (119.87–128.65)
LLA1 (1a)	74.94 (71.39–78.42.)	67.16 (62.66–71.74)	nd	nd	nd	nd	>400
LLA2 (2a)	300.6 (297.05–304.08)	287.26 (283.71–290.74)	nd	nd	nd	nd	>400
AmpB	0.27 (0.19–0.42)	0.065 (0.054–0.078)	0.022 (0.018–0.026)	nd	nd	nd	21.90 (18.23–26.87)
MA	nd	nd	nd	>400	>400	>400	>400

a *IC_50_, 50% Inhibitory Concentration*;

b *95% CI, Confidence interval*;

c *CC_50_: 50% Cytotoxic Concentration; nd, no determined; AmpB, amphotericin B; MA, meglumine antimoniate*.

### *In vitro* Cytotoxicity and Hemolytic Activities

LicoA (**1**) at concentrations evaluated exhibited moderate mammalian cytotoxicity against peritoneal murine macrophages, the CC_50_ values was 123.21 μM at 48 h. AmpB, exhibited mammalian cytotoxicity with CC_50_ values of 21.90 μM. On the other hand, MA showed no cytotoxicity at concentrations evaluated (Table 1). In the relationship between activity against intracellular amastigotes, and cytotoxicity in peritoneal murine macrophages, it was possible to determine that selectivity index (SI) of the LicoA (**1**) was 3.85 to *L (L.) amazonensis* and 4.16 to *L. (L) infantum* (data not shown). The hemolytic activity also was evaluated, and LicoA (**1**) showed about 30% of hemolytic activity at highest concentration evaluated (400 μM). On the other hand, AmpB showed 60% of hemolysis at concentration 100 μM and at higher concentrations (≥ 200 μM) was observed 100% of hemolysis.

### Treatment in Experimental Model of *Leishmania (L.) infantum*

**Figure 3:**
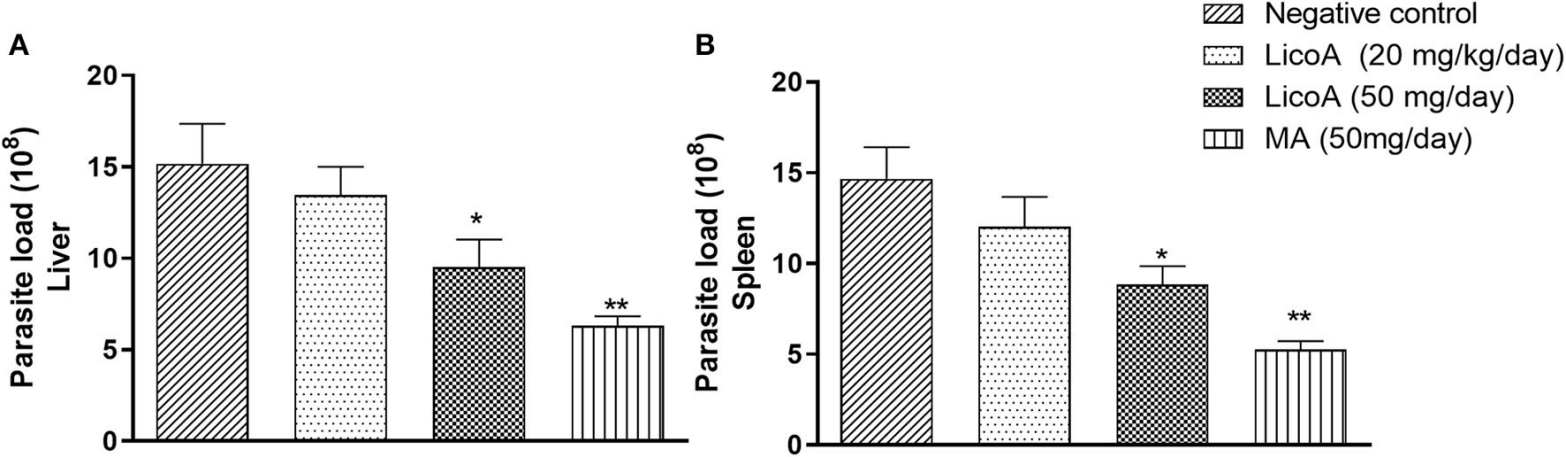
Liver **(A)** and spleen **(B)** parasite load of *L. (L.) infantum-*infected hamsters treated intraperitoneal with 20 and 50 mg/kg of body weight of LicoA (**1**) during eight consecutive days 4 weeks after infection. Thirty-five days after treatment the animals were sacrificed and the parasite burden was evaluated both in spleen and liver by limiting dilution method. Negative control group received the same number of injections of phosphate buffered saline (PBS) and animals from positive control group was treated with meglumine antimoniate (MA) (Glucantime®-Sanofi-Aventis) at dose 50 mg/kg of body. An asterisk indicates statistically significant differences as compared to the negative control group (PBS) (*p < 0.05, **p < 0.01).

**Figure 4:**
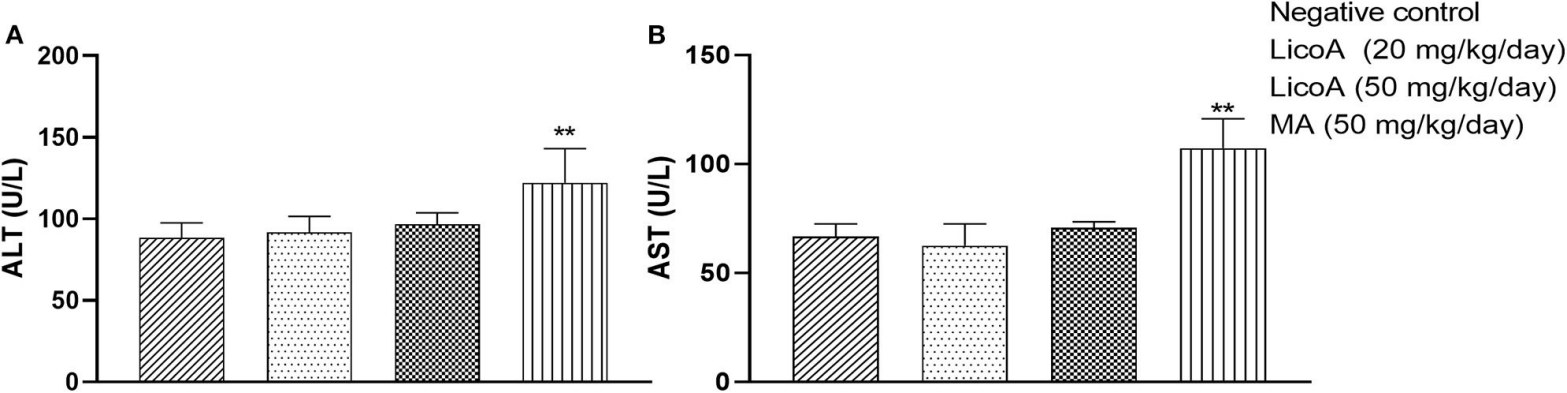
Serum concentrations of aminotransaminases ALT (alanine aminotransferase) **(A)** and AST (aspartate aminotransferase) **(B)** in no-infected hamsters and treated intraperitoneal with 20 and 50 mg/kg of body weight per day of LicoA (**1**) or treated with meglumine antimoniate (MA) (Glucantime®-Sanofi-Aventis) at dose 50 mg/kg of body weight per day during 15 days. Negative control group received the same number of injections of phosphate buffered saline (PBS) and animals from positive control group. An asterisk indicates statistically significant difference as compared to the negative control group (PBS) (**p < 0.01).

*L. (L.) infantum-*infected hamsters were treated during eight consecutive days at doses of 20 and 50 mg/kg of body weight per day of LicoA (**1**) 4 weeks after infection, and 35 days after treatment the animals were sacrificed. LicoA (**1**) at 20 and 50 mg/kg of body weight per day was able to significantly reduce the parasite burden in liver in 15.27 and 43.67%, respectively, and in spleen in 20.69 and 39.81%, respectively, when compared with negative control group. Positive control group that received 50 mg/kg of body weight per day of MA during eight consecutive days also showed significant reduction of parasite burden in 55.87% (liver) and of 63.57% (spleen) (Figures 3A,B). Besides, the average body weight animals showed no significant variation among negative, LicoA (**1**), and MA groups. However, after the treatment, 100% survival rate was registered in LicoA and MA groups, but in the negative group, the mortality of 33% of animals was observed (data not shown). Also, the animals no-infected and treated intraperitoneally at doses 20 and 50 mg/kg of body weight per day of LicoA (**1**) showed no significant alterations in the serum levels of aminotransaminases when compared with negative control group, but in the MA group, a significant increase in the serum levels of aminotransaminases was observed (Figures 4A,B).

## Discussion

Secondary metabolites from plants have been demonstrated promising leishmanicidal activity *in vitro* and *in vivo*, including compounds from chalcone class (8). Then, our decision to investigate LicoA (**1**) and its two analogs [**LLA1 (1a), LL2 (2a**)] was triggered by our previous study which demonstrated that LicoA (**1**) possess leishmanicidal activity against *L. (L.) major* and *L. (L.) donovani in vitro* and *in vivo*, which are parasites that cause CL and VL, respectively (10, 11). Besides, a search of the literature reports that LicoA (**1**) is of interest due to be inexpensive. Thus, this information encouraged us to evaluate the effect of LicoA and its two analogs against *L. (L.) amazonensis* and *L. (L.) infantum*, which are parasites involved in the DCL and CVL, respectively.

In this study, the synthesis of LLA1 and LLA2 was performed. As described, the prenylated chalcone LLA1 (**1a**) was prepared via the Claisen-Schmidt condensation between the prenylated vanillin and commercial p-methoxyacetophenone (Figure 2). The yield of this reaction was low; however, the difficulty in preparing prenylated chalcones was also found in another study that reports 16% yield for condensation of prenylated benzaldehydes (26). The analog LLA2 (**2a**) was obtained when the Claisen-Schmidt condensation between the prenylated vanillin and the *p*-methoxyacetophenone was performed using Lewis acid niobium pentachloride in dichloromethane. Niobium pentachloride promoted the formation of chalcone and hydrolysis of the prenyl group. Niobium pentachloride (NbCl_5_) has a wide variety of applications in organic synthesis, for example, in Diels-Alder reactions, multicomponent reactions, and polymerization reactions. In addition, niobium compounds are more readily available in Brazil due to the presence of the largest niobium reserves in the world (27). The obtained result shows that niobium pentachloride can be an efficient alternative for the synthesis of chalcones.

Among the compounds evaluated, LicoA (**1**) was the most promising compound against *Leishmania* parasites under the *in vitro* experimental conditions used. In general, compounds with IC_50_ values <10 μM *in vitro* in infective protozoan forms are considered as a hit and lead candidates for further *in vivo* evaluations (28). Thus, this study demonstrated that LicoA (**1**) possess moderate activity against amastigote forms of *L (L.) amazonensis* and *L. (L.) infantum*. On the other hand, previous studies demonstrated that LicoA (**1**) inhibits the *in vitro* growth of *L. (L.) major* and *L. (L.) donovani* promastigotes with IC_50_ value of ~0 μM for both parasites in 72 h, and a reduction in the number of amastigotes was observed at 2.5 μM after 72 h of incubation with LicoA (**1**) (10, 11). It should be noted that promastigote forms were more susceptible to LicoA (**1**) than amastigotes forms, and this effect can be attributed by distinct metabolism effects, as previous described in the literature (23).

By comparing the leishmanicidal activity observed to LicoA (**1**) with the positive control, the results demonstrated that promastigote and amastigote forms of *L (L.) amazonensis* were susceptible to AmpB. In contrast, promastigote and amastigote forms of *L. (L.) infantum* were not susceptible to the positive control, MA at conditions evaluated in this study. This results reinforces the hypothesis that pentavalent antimonials, such as MA act as prodrugs that require biological reduction to the trivalent to exert anti-leishmanial activity (29, 30). Although MA did not show leishmanicidal activity *in vitro*, this compound was choice to positive control *in vivo* assay, and then was evaluated under *in vitro* conditions. Additionally, we choice MA rather than sodium stibogluconate (SSG) because previous study has demonstrated that MA can be more effective than SSG in the treatment of leishmaniosis (31). As the experiments were performed in different periods, MA was not used as positive control against *L. (L.) amazonensis in vitroI*; however, pentavalent antimonials are considered first line agents in the therapy against leishmaniasis (6).

Despite of the moderate cytotoxic effect of LicoA (**1**) observed in our study, previous study has demonstrated that LicoA (**1**) shows low-toxicity in HFF cell *in vitro* (32). Also, study has demonstrated that LicoA (**1**) induces hemolysis to trigger cell shrinkage and phospholipid scrambling of the cell membrane at concentrations higher than 30 μM after 24 h (33).

Although the results *in vitro* have been better against *L. (L.) amazonensis*, the *in vivo* assay was performed against *Leishmania (L.) infantum* because this parasite is responsible by CVL, and also dogs are the main urban domestic reservoir of VL (3, 4). Several advances in the treatment of CVL have been made in the last years, but the pharmacotherapeutic options are still not satisfactory (5–7, 34). MA and allopurinol (alone or in combination) are drugs for CVL treatment and are able to achieve clinical cure (35). Other leishmanicidal drugs such as miltefosine in combination with allopurinol have also demonstrated leishmanicidal efficacy in infected dogs (36). Unfortunately, drug treatment cannot impede disease transmission since treated animals remain carriers of the parasite (5, 7, 34–36). Moreover, other aspects like toxicity and the rise of *Leishmania* strains that are resistant to the current use drugs highlights the importance of the development of new, safe and effective compounds capable to treat CVL and actually to eliminate the infection (5, 21, 34–36). Then, the *in vivo* activity of LicoA (**1**) was evaluated in experimental model of *Leishmania (L.) infantum* in hamster. The doses of LicoA (**1**) and the scheme of treatment were based on previous reports and also on dose of MA (11, 21). The results demonstrated a reduction of parasite burden in both liver and spleen to hamsters infected with *L. (L.) infantum* after treatment with LicoA (**1**). The positive control group, MA, showed better results than compared with LicoA (**1**), but both compounds significantly reduced the parasite burden. Previous study has shown that BALB/c mice infected with *L. (L.) major* and treated with LicoA (**1**) at doses 2.5 and 5 mg/kg of body weight by intraperitoneal administration 7 days after infection during 39 days completely prevented lesion development (11). Also, it was observed that the treatment of hamsters infected with *L. (L.) donovani* with intraperitoneal administration of LicoA (**1**) at a dose of 20 mg/kg of body weight per day for six consecutive days resulted in a >96% reduction of parasite load in the liver and the spleen compared with untreated control (11). However, our results demonstrated that at a dose of 20 mg/kg of body weight per day resulted in a reduction of parasite load of the 15.25 and 20.69% in liver and spleen, respectively. The intrinsic and/or acquired susceptibility of the parasite species can have contribute to differences of LicoA (**1**) susceptibility among the *Leishmania* species *in vitro* and *in vivo* when compared with the effect of LicoA (**1**) against *L. (L.) donovani* and *L. (L.) major* (10, 11). Previous study has demonstrated that intrinsic variation in miltefosine susceptibility of *Leishmania* clinical isolates was observed for visceral leishmaniasis in Nepal and cutaneous leishmaniasis in Peru (36). Another study demonstrated intraspecies differences in natural susceptibility to AmpB of clinical isolates of *Leishmania* subgenus *Viannia* (37). In this same study, it was demonstrated that isolates and strains maintained in the laboratory were less sensitive to AmpB when compared to clinical isolated (37).

No evidence of hepatotoxicity was found since serum concentrations of aminotransaminases showed no changed between LicoA-treated and non-treated groups. Previous study also demonstrated that LicoA (**1**) at doses higher than 1,000 mg/kg, administered orally once a day for 2 weeks did not cause any signs of toxicity. Also, intraperitoneal administration of LicoA (**1**) at doses of 100 mg/kg in rats and 150 mg/kg in hamsters did not show any toxicity (11). In addition, study has been shown that LicoA (**1**) has a protective effect against Acetaminophen overdose-induced hepatotoxicity (38). These findings suggest the safety of LicoA (**1**) in animal model independently of the route of administration. Notwithstanding positive control group (animal treated with MA) has showed better results than compared with LicoA (**1**) at dose 50 mg/kg of body weight per day, pentavalent antimonials cause serious side effects (4, 7, 8). Also, the MA administration can not be administered by the oral route due irritation of intestinal mucosa (39), but LicoA (**1**) could be evaluated by the oral route in the future research in *L. (L.) infantum-*infected hamsters.

About the action mechanism, studies suggest that the effect of LicoA may be due to the altering of both ultrastructure and function of the mitochondria of *Leishmania* parasites (40). Moreover, studies involving *in silico* approaches also reinforced the antileishmanial potential of LicoA by denoting its capability to inhibit the NADPH-dependent fumarate reductase, which is the key enzyme in the fumarate respiration. This anaerobic process is essential for the growth and survival of the parasite in their host, but is absent in the normal cells of the mammalian hosts (41, 42). In addition, studies of the modeling and docking suggest that LicoA is a potent inhibitor of cysteine proteases A, which are the essential virulence factors in leishmania parasitic (43–45). Also, study has been shown that chalcones act against malarial papain-like cysteine proteases, which is an important mechanism of the hemoglobin degradation (46).

Given in consideration that CVL manifests through splenomegaly possibly associated with hepatomegaly, and keeping in mind the lack of hepatoxicity of LicoA (**1**) and its *in vitro* effect against *L (L.) amazonensis* and *L (L.) infantum*, and *in vivo* effect in hamster-infected with *L (L.) infantum*, further studies to address the effects LicoA (**1**) and other chalcones on pathological changes associate to leishmaniosis are crucial. In addition, the results suggest that chalcone-type flavonoids are a promising class of natural products to be considered in the search of new compounds capable to treat CVL, and the association of such compounds with nanostructured carriers may lead to formulations that can increase the efficacy, selectivity, and safety of the CVL treatment.

## Data Availability Statement

The raw data supporting the conclusions of this article will be available by request from the corresponding author.

## Ethics Statement

The experiments were conducted in accordance with the Brazilian legislation regulated by the National Council for the Control of Animal Experimentation (CONCEA) and approved by the University of Franca's Ethics Committee for Animal Care under protocol number 046/15 (Approval Date: November, 09, 2015).

## Author Contributions

JS, EC, AC, MF, RM, and FD carried out the biological experiments and maintenance of *Leishmania* parasites. RP, AN, and MC contributed to the isolation, synthesis, and characterization of the compounds. SA, AS, and MS supervised the chemical part of the study. AA, LM, and RV supervised the work and provided the facilities for biological and chemical activities. All authors contributed to the article and approved the submitted version.

## Conflict of Interest

The authors declare that the research was conducted in the absence of any commercial or financial relationships that could be construed as a potential conflict of interest.
